# The Ubiquitin–26S Proteasome Pathway and Its Role in the Ripening of Fleshy Fruits

**DOI:** 10.3390/ijms24032750

**Published:** 2023-02-01

**Authors:** Wen Jia, Gangshuai Liu, Peiyu Zhang, Hongli Li, Zhenzhen Peng, Yunxiang Wang, Tomislav Jemrić, Daqi Fu

**Affiliations:** 1 Laboratory of Fruit Biology, College of Food Science and Nutritional Engineering, China Agricultural University, Beijing 100083, China; 2 Institute of Agri-Food Processing and Nutrition, Beijing Academy of Agriculture and Forestry Sciences, Beijing 100097, China; 3 Department of Pomology, Division of Horticulture and Landscape Architecture, Faculty of Agriculture, University of Zagreb, 10000 Zagreb, Croatia

**Keywords:** 26S proteasome, ubiquitination, degradation, ripening of fleshy fruit

## Abstract

The 26S proteasome is an ATP-dependent proteolytic complex in eukaryotes, which is mainly responsible for the degradation of damaged and misfolded proteins and some regulatory proteins in cells, and it is essential to maintain the balance of protein levels in the cell. The ubiquitin–26S proteasome pathway, which targets a wide range of protein substrates in plants, is an important post-translational regulatory mechanism involved in various stages of plant growth and development and in the maturation process of fleshy fruits. Fleshy fruit ripening is a complex biological process, which is the sum of a series of physiological and biochemical reactions, including the biosynthesis and signal transduction of ripening related hormones, pigment metabolism, fruit texture changes and the formation of nutritional quality. This paper reviews the structure of the 26S proteasome and the mechanism of the ubiquitin–26S proteasome pathway, and it summarizes the function of this pathway in the ripening process of fleshy fruits.

## 1. Introduction

Proteins, including structural, enzyme, regulatory, and receptor proteins, etc., are crucial to all aspects of life. To maintain dynamic equilibrium, an organism continuously produces and degrades protein. Protein degradation pathways are mainly divided into two categories, energy dependent and energy independent [1]. Among them, the energy-dependent ubiquitin–26S proteasome pathway is the main one. In eukaryotes, the 26S proteasome is a significant protein complex, and it is made up of several subunits. It is widely distributed throughout the cytoplasm and nucleus. Additionally, it has a proteolytic action, mostly responsible for degrading damaged and improperly folded proteins in cells as well as a few regulatory proteins, so as to take part in a number of physiological processes [2,3]. The structure and function of the 26S proteasome is highly conserved in eukaryotes. The ubiquitin–26S proteasome pathway is a typical post-translational regulation of proteins [4].

Ubiquitin (Ub), a polypeptide with 76 amino acids that are remarkably conserved in sequence, exist in all eukaryotes [5]. It can bind to proteins in cells by covalent bonds [6]. The substrate protein of the ubiquitin–26S proteasome pathway requires ubiquitination modification before it can be recognized. The ubiquitin–26S proteasome pathway ensures efficient degradation of protein and substrate specificity via substrate ubiquitination and the fine structure of proteasomes [7]. Numerous aspects of plant growth and development, including the cell cycle, cell division, and differentiation, as well as defense and stress responses, hormone signal transduction, etc., depend on the ubiquitin–26S proteasome system [8,9]. In recent years, the ubiquitin–26S proteasome pathway has also gradually received researchers’ attention in fruit development and ripening. Fruit ripening is a crucial stage in the formation of fruit quality and is a complex biological process. It involves numerous physiological and biochemical changes, including the biosynthesis and signaling of related hormones, pigment accumulation, fruit softening, nutrient formation, etc. These changes are primarily brought about by the selective expression of genes related to fruit ripening and are also closely linked to the synthesis and degradation of the proteins that these genes encode [10]. The entire ripening process for fleshy fruits involves the ubiquitin–26S proteasome pathway, which can take part in the regulation of transcription factors, receptor proteins, and other proteins. This paper reviews the structure of the 26S proteasome and the mechanism of the ubiquitin–26S proteasome pathway, and it summarizes the function of the ubiquitin–26S proteasome pathway in the ripening process of fleshy fruits.

## 2. Structure of the 26S Proteasome

**Figure 1:**
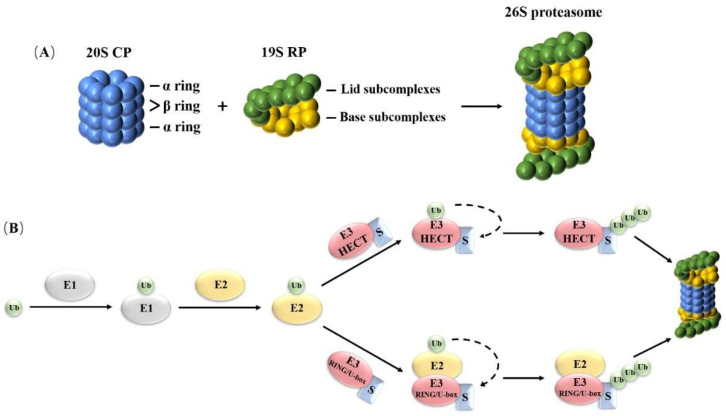
Structure of the 26S proteasome and the ubiquitin pathway. (**A**) 20S CP (20S Core Particle); 19S RP (19S Regulatory Particle). (**B**) Ubiquitination pathway. Ub, ubiquitin; E1, ubiquitin-activating enzyme; E2, ubiquitin-conjugating enzyme; E3 HECT, ubiquitin ligase enzyme with homologous to E6-associated protein C-terminus domain; E3 RING/U-box, ubiquitin ligase enzyme with really interesting new gene/U-box domain; S, substrate protein.

The 26S proteasome is an ATP-dependent proteolytic complex, mainly composed of 20S core particle (CP) and 19S regulatory particle (RP). The 19S RP is attached to both ends of the core particle, as shown in Figure 1A.

The 20S CP, also known as 20S proteasome, has stable biochemical properties and can exist independently of the 26S proteasome [11]. The 20S CP is barrel shaped and consists of 4 rings (2 α rings and 2 β rings). The α ring is located on the outside and is composed of 7 α subunit named PA (A–G). The β ring is located inside and is composed of 7 β subunit named PB (A–G). The order of the four rings of 20S CP is α-β-β-α [12,13]. The 20S CP forms a closed cavity inside, providing space for protein degradation. Its protein degradation activity is determined by 3 β subunits in the β ring. β1 (PBA) has peptidyl glutamyl peptidase activity, β2 (PBB) has tryptase-like hydrolysis activity, and β5 (PBE) has chymotrypsin-like activity [12]. During the assembly of 20S CP, the active site of threonine was exposed, which enabled the proteasome to cut most peptide bonds of target proteins [14]. The N-terminal of each subunit of the α ring extends outward to point to the center of the ring, forming a narrow channel that allows only the unfolded substrate protein to enter and restricts the entry of other proteins [15,16]. In the 26S proteasome, this channel is regulated by the ATPase subunit at the bottom of 19S RP and further stimulates the protease activity of 20S CP [17].

The 19S RP, which is located at both ends of the 20S CP, is composed of two parts: base subcomplexes and lid subcomplexes [18]. The base subcomplexes contains three non-ATPase subunits (named Rpn1, Rpn2, Rpn13 in Saccharomyces cerevisiae) and six ATPase subunits (Rpt1–6). Rpn1 and Rpn13, as ubiquitin receptors, provide multiple binding sites for ubiquitin and ubiquitin-like proteins (UBLs). Among them, Rpn13 is attached to the 19S RP by combining with Rpn2 [19,20,21]. Six ATPase subunits could polymerize to form a cyclic heterohexamer through the AAA+ domain, which can combine with the substrate protein, use the energy generated by ATP hydrolysis to unfold the folded protein, and then transfer the unfolded polypeptide to 20S CP for degradation [22,23]. The lid subcomplexes consists of nine non-ATPase subunits, which are responsible for recognizing ubiquitin degradation signals and substrate protein deubiquitination [8]. Among them, Rpn11 is a deubiquitinase (DUB). As a functional subunit that performs deubiquitination, Rpn11 is responsible for the removal of the substate-attached ubiquitin chains before they enter the ATPase subunits polymer [8,24]. In addition to the 19S RP, there is also a subunit Rpn10, which is an ubiquitin receptor. When the 26S proteasome performs its function, Rpn10 can directly interact with the lid subcomplexes subunits Rpn8 and Rpn9 in 19S RP [25]. The 26S proteasome also contains one or two stable bound DUBs, namely, Ubp6 and Uch37. Ubp6 (named Usp14 in mammals) is an ubiquitin specific protease, which is used to cut the redundant ubiquitin chain in the substrate protein [26]. In comparison with Saccharomyces cerevisiae, there is another Uch37 (also known as UCHL5) subunit in the human body, which exists as ubiquitin C-terminus hydrolase and may be used to cut or edit the distal ubiquitin chain of substrate protein [27]. The synergistic effect of the lid subcomplexes and base subcomplexes ensures that the ubiquitinated protein is recognized and transferred to the core particles for degradation.

## 3. Mechanism of the Ubiquitin–26S Proteasome Pathway

### 3.1. Ubiquitination of Substrate Protein

The participation of ubiquitin, ubiquitin-activating enzyme (E1), ubiquitin binding enzyme (E2), and ubiquitin ligase enzyme (E3) is necessary for the ubiquitination of substrate protein [28]. Among them, E3 have many kinds of functions. According to the difference in their domains, E3 ubiquitin ligases in plants can be divided into three categories, including Homologous to E6-associated protein Carboxyl terminus (HECT), Really interesting new gene (RING)/U-box, and RING-in-between-RING (RBR) [29,30,31,32,33], and all of them can mediate the ubiquitination of substrate proteins. RING/U-box E3 ubiquitin ligases can be divided into two types: single-subunit and multi-subunit E3 ligases. The single-subunit RING/U-box E3s include Constitutive Photomorphogenesis 1 (COP1), SEVEN IN ABSENTIA IN ARABIDOPSIS THALIANA 5 (SINAT5) and Arm Repeat-Containing 1 (ARC1) proteins; the multi-subunit RING/U-box E3s include SCF (SKP1, Cullin1 and F-box proteins), CUL3-BTB (Cullin3, BTB and RBX1 proteins) and Anaphase Promoting Complex (APC) [29]. Existing studies show that SCF complex is a major E3, and F-box protein and cullin protein are important components of SCF complex, because they can bind to substrate protein [34]. In particular, the cullin proteins usually act as the scaffold of the complex. They can interact with specific substrate receptors via the N-terminal sequence, and they can bind RBX1 (RING BOX 1) via the C-terminal domain so as to recruit the E2 to form the E3 ligases, which can also be called Cullin-RING Ligases (CRLs) [31,35]. In *Arabidopsis*, there are four main cullin types (CUL1, CUL2, CUL3A/B, and CUL4), and they have been shown to be the components of CRLs [32,36]. Phylogenetic analysis in wheat and rice also showed that the cullin protein family includes four clades, cullin 1~cullin 4 [37,38].

As shown in Figure 1B, in the substrate protein ubiquitination pathway, firstly, using the energy provided by ATP, the carboxyl group at the C-terminus of ubiquitin molecule covalently connects with the cysteine residue of the active center of E1 to form an E1–ubiquitin complex through a high-energy thioester bond, resulting in the activation of ubiquitin. E2 contains conservative cysteine residues. Through ester exchange with the E1–ubiquitin complex, the activated ubiquitin binds to E2 to form an E2–ubiquitin complex. E3 can transfer ubiquitin in two ways: one is that the E3, containing HECT domain, directly interacts with ubiquitin, receives ubiquitin molecules in the E2–ubiquitin complex, and then uses E3 as a donor to transfer ubiquitin to the substrate protein; the other way is that the E3, containing RING/U-box domain, directly binds to the E2–ubiquitin complex, promoting E2 to transfer ubiquitin to the substrate protein [29,30]. Glycine at the C-terminus of ubiquitin molecule can be covalently linked with lysine at the side chain of substrate protein through heteropeptide bond, and glycine at the C-terminus of other ubiquitin molecules can be further linked with lysine at the side chain of ubiquitin, thus forming the polyubiquitin chain [30,31,39]. The polyubiquitin chain can promote the process of the protein degradation, but it does not play the role of degradation itself [5]. For different substrates or different degradation purposes, the length, position and specificity of ubiquitin chains formed in the proteins are different, so their proteasome affinity is also different [40].

### 3.2. Recognition of Ubiquitinated Substrate Protein

The initial binding of ubiquitinated substrate protein to the 26S proteasome depends on the polyubiquitin chain [41]. The polyubiquitin chains contain many types of linkage, such as K48-linked chain, and they can act as the signal for the 26S proteasome to recognize and target the substrate, and deliver them the 26S proteasome [5]. There are three ubiquitin receptors in the 26S proteasome, Rpn1, Rpn10 and Rpn13, among which Rpn10 and Rpn13 play a major role in the initial binding with ubiquitinated proteins [2,8]. It was found that the Rpn1 receptor is mainly recognized by two circular repeats with α-helical grooves that bind to ubiquitin. Rpn10 receptor binds to ubiquitin chain via ubiquitin interacting motif (UIM), and Rpn13 receptor binds to ubiquitin chain via Pleckstrinlike Receptor for Ubiquitin (PRU) domain [42,43]. Moreover, Rpn1, Rpn10 and Rpn13 can also be used as receptor indirect binding substrates containing ubiquitin-like (UBL) domain and ubiquitin-associated (UBA) domain proteins [2,44]. Rpn1, Rpn10 and Rpn13, as the multi-functional structural basis in the 26S proteasome, allow the recognition of substrates with different conformations and ubiquitin chains with different lengths and connection modes, but their synergistic effects are still unclear [44]. The tight combination of ubiquitinated substrate protein and the 26S proteasome requires the substrate to form a loose folding region by energy provided by ATP hydrolysis [41]. Ubiquitinated substrate proteins can not only be recognized by Rpn1, Rpn10 and Rpn13, but some exogenous receptors outside the 26S proteasome can also transfer substrate proteins through interactions with proteasome and ubiquitin chains [8].

### 3.3. The 26S Proteasome Deubiquitination

After the ubiquitinated protein is recognized by the 26S proteasome, the polyubiquitin chain will be released through the DUBs, and the free ubiquitin can be reused. In eukaryotes, there are mainly three kinds of DUBs including metalloproteinase Rpn11, cysteine protease Ubp6 (named Usp14 in mammals) and Uch37/UchL5 [8,45].

Rpn11 is the most important DUB in the 26S proteasome [8]. Rpn11 is located above the 20S CP, and it can scan the substrate comprehensively before the substrate enters the degradation channel, so as to avoid the substrate’s premature deubiquitination [46]. Rpn11 completely eliminates ubiquitin modification by hydrolyzing the heteropeptide bond between the lysine of substrate protein and the C-terminus of the first ubiquitin [47]. Ubp6/Usp14 is a ubiquitin-specific protease (USP), and it can work only when multiple ubiquitin chains are attached to the substrate. Its USP domain at the C-terminus has catalytic activity of cutting the redundant ubiquitin chains in the substrate protein. Additionally, its UBL domain at the N-terminus can interact with Rpn1 in 19S RP, which also significantly enhanced Ubp6′s ubiquitination activity [26,48]. After binding to ubiquitin, Ubp6 can induce the conformational change in the 26S proteasome, which activates ATPase subunit to open the degradation channel of 20S CP [8,48]. Uch37, also known as UCH-L5, is a cysteine-dependent DUB, and it can bind to the ubiquitin receptor Rpn13 to cut or edit the distal ubiquitin chain of the substrate protein [8,27,49]. The research of Peth et al. [50] reveals that Uch37 can also activate ATPase subunits and promote 20S CP to open the degradation channel, which indicates that it can affect the conformation of proteasome just like Ubp6. Rpn11, Ubp6 and Uch37 can independently perform their functions and degrade the substrate protein ubiquitin chain in different ways which depend on the length, number and connection type of the ubiquitin chain. Moreover, Ubp6 can interact with Rpn1 in 19S RP to interfere with ubiquitin binding to Rpn11 [8].

Eukaryotes also contain other DUBs, such as Usp5, Usp7, Usp13, etc. It has been found that they are also related to the ubiquitin–26S proteasome pathway [51]. For example, the large accumulation of free ubiquitin chains will interfere with the activity of the 26S proteasome, resulting in the inhibition of the degradation of some substrate proteins. Usp5 can selectively hydrolyze free ubiquitin chains to ensure that proteasome performs its normal function [52]. In general, the basic function of all DUBs is to release ubiquitin molecules to promote the degradation of substrate proteins or promote the recycling of ubiquitin, and studies have found that the specificity of proteasome can be adjusted by rapidly removing ubiquitin chains [26].

### 3.4. The 26S Proteasome Degrades Substrate Protein

The 26S proteasome can efficiently degrade substrate proteins through precise conformation regulation. In this process, the combination of DUBs and ubiquitinated proteins can induce significant changes in the structure of 19S RP. When ATP binds to the ATPase subunit in 19S RP, the channel of the hexamer ATPase loop will expand. At this time, the N-terminus of each subunit of the α ring in the 20S CP extends outward to the center of the ring, thus forming a channel that leads to the 20S CP [50,53]. The deubiquitinated substrate protein can linearize their folding domain through the mechanical force generated by the ATPase subunit. The pore ring formed by six central tyrosines is responsible for unfolding the substrate protein to drive the substrate translocation [54,55]. After the substrate protein transposed in a certain direction, it can be recognized by specialized peptidase in 20S CP, and then be transmitted and cut, thus realizing the degradation of the substrate protein. Among them, the channels formed between the subunits of the α ring in 20S CP can allow the entry of substrate proteins after their removal of ubiquitin chains, and also can prevent the non-selective entry of other cell proteins, thus ensuring the precision of the ubiquitin–26S proteasome pathway [16].

In this process, the deubiquitination of substrate and protein hydrolysis driven by ATP are the two most critical steps, which can be closely linked through Usp6/Ubp14 and Uch37 [8,50]. Usp6/Ubp14 and Uch37 can not only interact with ubiquitinated substrate proteins, but also activate ATPase subunits in 19S RP. When ATP binds to the ATPase subunits, the HbYX motif at the C-terminus of the two ATPase subunits, Rpt2 and Rpt5, is the key to promote substrate protein degradation. HbYX is a conservative C-terminus motif, in which Hb is a hydrophobic residue and Y refers to tyrosine (phenylalanine in some archaea), and X can change. Some studies have demonstrated that only the C-terminus of ATPase subunit with the HbYX motif can cause α subunit rotation, and then lysine binding to α subunits induces 20S CP to open the degradation channel [56,57]. However, the ATPase subunit Rpt3 is an exception. Although Rpt3 also contains the HbYX motif, it has not been confirmed that it can induce 20S CP to open the channel. The other three ATPase subunits in 19S RP, including Rpt1, Rpt4 and Rpt6, do not contain the HbYX motif. Although Rpt1 can weakly induced channels opening under certain conditions, Rpt1 do not perform similar functions to Rpt3 but can maintain the binding between 19S RP and 20S CP [57]. Although the six ATPase subunits play different functions, the loss of any ATPase subunit will lead to the loss of most activities of ATPase subunit, which indicates that the protein degradation pathway requires the coordination of all Rpt subunits. Peth et al. [58] found that different ubiquitinated substrates can activate ATPase subunits to drive protein hydrolysis, and the degradation rate of ubiquitinated proteins is closely coupled with ATP hydrolysis rate. It can be seen that the activity of ATPase subunits plays an important role in the binding, unfolding and translocation of substrate proteins, and is closely related to the activity of the whole 26S proteasome.

## 4. Function of the Ubiquitin–26S Proteasome Pathway in Fleshy Fruit Ripening

In plants, the ubiquitin–26S proteasome, as a way of post-translational regulation, is an important pathway of protein degradation. The range of substrate proteins of the ubiquitin26S proteasome pathway is very wide, involving almost all aspects of the plant life cycle, such as cell signal transmission, plant growth and development, circadian rhythm, biological and abiotic stress, cell apoptosis, etc. [8,59]. Our understanding of the function of the ubiquitin–26S proteasome pathway is still limited, especially its function during the ripening of fleshy fruits [60,61]. Fleshy fruit ripening is a complex biological process, during which fruit ripening-related genes are selectively expressed [62]. The abundance of proteins encoded by these genes is regulated by the ubiquitin–26S proteasome pathway. This review will focus on the role of the ubiquitin–26S proteasome pathway in the process of fleshy fruit ripening from four aspects: biosynthesis and signal transduction of hormones related to fleshy fruit ripening, pigment metabolism, texture change and nutrient quality formation.

### 4.1. The Ubiquitin–26S Proteasome Pathway Is Involved in Regulating Biosynthesis and Signal Transduction of Hormones Related to Fleshy Fruit Ripening

Fruit development and ripening are regulated by plant hormones [63]. Studies have revealed that the ubiquitin–26S proteasome pathway mediates the regulation of ethylene, auxin and abscisic acid, especially the F-box protein [64,65,66,67].

#### 4.1.1. Ethylene

**Figure 2:**
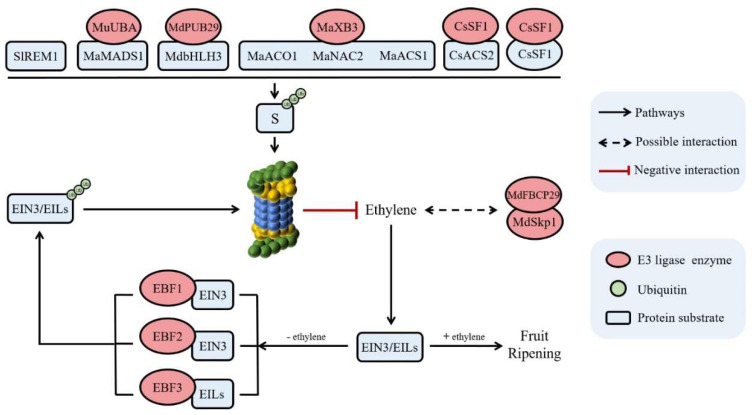
The function of the ubiquitin–26S proteasome pathway in ethylene biosynthesis and signal transduction pathway. S: substrate proteins, such as SlREM1, MaMADS1, MdbHLH3, MaACO1, MaNAC2, MaACS1, CsACS2, and CsSF1.

Fleshy fruits can be divided into climacteric fruits and non-climacteric fruits according to whether there is a respiratory peak during ripening. Among them, ethylene is a necessary condition for the initiation of climacteric fruit ripening, and there is an obvious peak of respiratory ethylene during their ripening [68], while the ripening process of non-climacteric fruit does not depend on ethylene [69]. The ubiquitin–26S proteasome pathway plays an important regulatory role in ethylene biosynthesis and signal transduction, as depicted in Figure 2. ACC synthase (ACS) and ACC oxidase (ACO) are key enzymes in ethylene biosynthesis pathway [68]. Kim et al. [70] found that the ubiquitin–26S proteasome pathway is involved in the degradation of ACS protein in the wounded tomato fruit. Moreover, there is also a membrane protein Remorins 1 (SlREM1) in tomato fruit, which can interact with ethylene biosynthetic protein, such as S-adenosylmethionine synthetase 1 (SAM1), ACO1 and ACS2, to promote ethylene biosynthesis. The ubiquitin–26S proteasome pathway can mediate the degradation of SlREM1 protein, thereby negatively regulating ethylene biosynthesis and fruit ripening [71]. By degrading itself and ACS2 through ubiquitination, the *Short Fruit 1* (*SF1*) gene, which codes for a melon-specific RING-type E3 ubiquitin ligase, can decrease ethylene synthesis, which in turn affects cell division and fruit elongation [72]. MaXB3, a RING-type E3 ubiquitin ligase in banana, can interact with MaACS1 and MaACO1 and specifically mediates their degradation through the ubiquitin–26S proteasome pathway, thereby resulting in the inhibition of ethylene biosynthesis [73]. MdFBCP1 protein contains the F-box domain in apples, and it can interact with MdSkp1 to form the SCF complex, and then function as E3 ubiquitin ligase [74]. *MdFBCP1* is highly expressed in mature climacteric fruits and is induced by ethylene, which is similar to that of the ethylene-inducing genes *MdACO1* and *MdCAS2* in apples. Therefore, the expression of *MdFBCP1* may regulates ethylene-induced apple fruit ripening [74]. The MdPUB29 in apple fruits is a U-box E3 ubiquitin ligase, and it can target and degrade MdbHLH3 transcription factors by the ubiquitin–26S proteasome pathway to inhibit the expression of ethylene biosynthesis related genes *MdACO1*, *MdACS1* and *MdACS5A*, resulting in the suppression of fruit ripening [75]. In banana fruit, the transcription factor MaMADS1 related to fruit ripening interacts with the E1 ubiquitin-activating enzyme MuUBA and was degraded through the ubiquitin–26S proteasome pathway, affecting fruit ripening [76].

Ethylene can also regulate fruit ripening via the signal transduction pathway [77]. A series of elements of the ethylene signal transduction pathway have been reported in plant, such as ethylene receptor (ETR), CONSTITUTIVE TRIPLE RESPONSE 1 (CTR1), ETHYLENE INSENSITIVE 2 (EIN2), ETHYLENE INSENSITIVE 3 (EIN3)/EIN3-like proteins (EILs), and ethylene response factors (ERFs) [78]. EIN3/EILs are key transcription factors in ethylene signal transduction, and their protein levels are regulated by the ubiquitin–26S proteasome pathway. In the absence of ethylene, EIN3 was rapidly ubiquitinated, and then degraded by the 26S proteasome. However, in the presence of ethylene, the ubiquitination degradation of EIN3 is inhibited, and EIN3 accumulates in a large amount in the nucleus, activating ethylene reaction [79,80]. Studies have shown that the SCF (SKP1-CUL1-F-box Rbx1) complex plays a role as an E3 ubiquitin ligase in the regulation of the ubiquitin–26S proteasome pathway of ethylene signal transduction, in which two F-box proteins, EBF1 and EBF2 (EIN3 Binding F-box protein 1 and 2), are responsible for recognizing EIN3, and then mediating the ubiquitination degradation of EIN3 [65,81]. Yang et al. [82] identified two F-box genes in the tomato EBF subfamily: *SlEBF1* and *SlEBF2*. The F-box protein EBF1/2 can interact with the EIN3 protein, thus mediating its ubiquitination degradation. This negatively regulates the ethylene signal pathway and inhibits tomato fruit ripening. Another F-box protein in tomato fruit, SlEBF3, can interact with EILs and induce their ubiquitination degradation, inhibiting ethylene signal transduction and fruit ripening [83]. Zhao et al. [84] cloned the *EIN3* gene and ubiquitinated components *PuEBF1* and *PuEBF2* in Nanguo pear. The results of yeast two-hybrid and pull-down tests demonstrated that PuEIN3 could interact with PuEBF1 and PuEBF2, providing evidence for PuEIN3 to be degraded by ubiquitination. An F-box gene *MdEBF1* has also been identified in apples. Further research shows that MdEBF1 can negatively regulate the expression of softening-related gene *POLYGALACTURONASE1* (*PG1*) by inhibiting the activity of EILs, which indicates that MdEBF-like protein may play an important role in ethylene mediated fruit ripening [85]. In addition, Shan et al. [73] also found that MaXB3 can also mediate the ubiquitination degradation of the transcription factor MaNAC2 (NAM, ATAF, and CUC 2), thereby reducing the transcriptional inhibition of MaNAC2 on the ethylene biosynthesis inhibitor *MaERF11*, and negatively regulating fruit ripening.

#### 4.1.2. Auxin

Auxin is mainly responsible for regulating cell division, cell expansion and cell differentiation [86]. The ubiquitin–26S proteasome pathway can participate in regulating fruit ripening in an auxin-dependent way. Auxin response factors (ARFs) and auxin/indole-3-acetic acid inhibitors (Aux/IAA) mediate auxin signaling [87,88]. In the case of auxin deficiency, Aux/IAA and ARFs can directly assemble into dimers through their conserved domains, preventing ARFs from functioning, thereby inhibiting the activation of auxin response genes [89,90]. When a high level of auxin exists, it will promote the combination of F-box auxin receptor protein TRANSPORT INHIBITOR RESPONSE 1/AUXIN SIGNALING F-BOX (TIR1/AFB) and SKP1-like proteins to form the E3 complex SCF^TIR1^/AFB, which can target the degradation of transcription inhibitors Aux/IAAs through the ubiquitin–26S proteasome pathway, release the ARFs, and further regulate the transcription level of its downstream auxin response genes [91,92,93,94]. Islam et al. [95] isolated and identified three F-box auxin receptors in plum fruits, PslTIR1, PslAFB2 and PslAFB5. Overexpression of *PslTIR1* in tomato (*Solanum lycopersicum*) fruits can amplify the regulatory effect of IAA on fruit maturity, indicating that TIR1 mediated the ubiquitin–26S proteasome pathway may regulate fruit maturity by activating IAA signal transduction [96].

#### 4.1.3. Abscisic Acid

Abscisic acid (ABA) plays an important role in seed dormancy and germination, stomatal movement, fruit development, stress response, etc., and it can promote the ripening process of climacteric and non-climacteric fruits [97,98,99]. Multiple signal transduction elements of ABA signal transduction pathway are regulated by ubiquitination [100]. Yu et al. [101] found that the E3 ubiquitin ligase VlPUB38 in strawberry fruit can interact with abscisic aldehyde oxidase (VlAAO), a key factor in the abscisic acid biosynthesis pathway, depending on its U-box conservative domain, and target the degradation of VlAAO protein through the ubiquitin–26S proteasome pathway, negatively regulating strawberry fruit ripening. Tan et al. [102] identified, classified and characterized the E3 gene of whole genome of peach (*Prunus Persica*) fruit, and found that *PpPUB9* and *PpATL43* are homologous to genes involved in ABA signal transduction pathway, such as *AtPUB9* and *AtATL43*, which mediate ubiquitination of ABA receptors [100]. However, the role of these E3 genes in peach fruit ripening requires further study.

### 4.2. The Ubiquitin–26S Proteasome Pathway Is Involved in Regulating Pigment Metabolism of Fleshy Fruits

**Figure 3:**
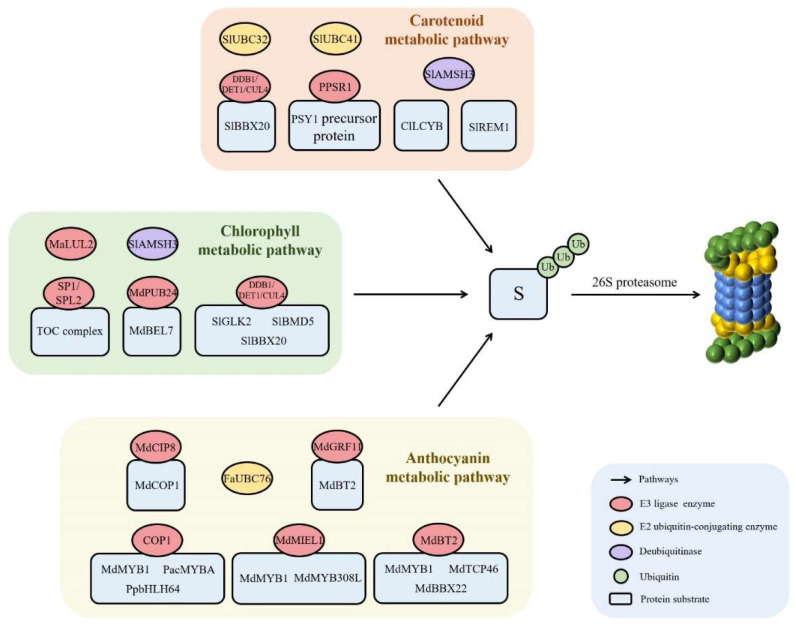
The function of the ubiquitin–26S proteasome pathway in pigment metabolism. S: substrate proteins, such as SlBBX20, PSY1 precursor protein, ClLCYB, SlREM1, TOX complex, MdBEL7, SlGLK2, SlBMD5, MdCOP1, MdBT2, MdMYB1, PacMYBA, PpbHLH64, MdMYB308L, MdTCP46, and MdBBX22.

During the ripening of fleshy fruits, the color of fruits will change, such as the loss of chlorophyll in chloroplasts, the accumulation of carotenoids in colored bodies, and the accumulation of flavonoids (such as anthocyanins) [103,104]. The pigment metabolism pathway is affected by many factors such as light, temperature, hormones, and expression level of related genes. At the protein level, pigment metabolism is also regulated by the ubiquitin–26S proteasome pathway [103], as shown in Figure 3.

The transformation from chloroplast to chromoplast is one of the most significant characteristics during fruit ripening, which involves the degradation of chlorophyll, destruction of thylakoids and accumulation of carotenoids [105]. The suppressor of ppi1 locus 1 (SP1), a RING-type E3 ubiquitin ligase, is located in the outer membrane of plastids. The discovery of SPL1 in tomato fruit reveals that the ubiquitin–26S proteasome pathway can regulate fruit ripening by selectively targeting plastid protein degradation [105]. Ling et al. [106] found that knockout of *SPL1* or its homologue *SPL2* in tomatoes can delay fruit ripening, and overexpression of *SPL1* will accelerate fruit ripening, which indicates that SPL1 can promote the color transformation during fruit maturity by promoting the transformation from chloroplast to colored body. The study found that E3 ubiquitin ligase based on the CUL4 protein plays an important role in regulating the plastid level and pigment accumulation of tomato fruit [107]. The UV-damaged DNA binding protein 1 (DDB1) and de-etiolated-1 (DET1) homologous proteins encoded by *high pigment 1* (*hp1*) and *high pigment 2* (*hp2*) in tomato fruits are important components of the CUL4-type E3 ubiquitin ligase. The CUL4-DDB1-DET1 complex formed by them can regulate the plastid level of tomato fruits by targeting the transcription factors Golden 2-Like (SlGLK2) and B-box protein 20 (SlBBX20), then regulate pigment accumulation [107,108,109]. In addition, methylation recognition protein methyl-CpG binding domain 5 (SlMBD5) can synergistically regulate pigment metabolism by interacting with CUL4-DDB1-DET1 complex [110]. In apple fruit, the U-box E3 ubiquitin ligase MdPUB24 interacts with the transcription factor MdBEL7 and ubiquitinates it, and then degrades it through the ubiquitin–26S proteasome pathway. The degradation of MdBEL7 can eliminate the transcriptional inhibition of genes related to downstream chlorophyll degradation, leading to chlorosis of apple fruits during storage [111]. A RING-type E3 ubiquitin ligase MaLUL2 identified by Wei et al. [112] in banana fruits also participate in the degradation of chlorophyll. The study found that the transient overexpression of *MaLUL2* in banana peel can increase the ubiquitination level in banana peel, negatively regulate the chlorophyll degradation during banana fruit development, and maintain the green phenotype of the banana fruit. Wang et al. [113] found that PSY1 protein, a key rate limiting enzyme in the carotenoid biosynthesis pathway in tomatoes, was ubiquitinated through interaction with the RING-type E3 ubiquitin ligase Plastic Protein Sensing RING E3 ligase 1 (PPSR1), and it was further degraded through the 26S proteasome, resulting in the regulation of the accumulation of carotenoids by affecting its stability in tomato fruits. Zhang et al. [114] found that lycopene β cyclinase, encoded by the *ClLCYB* gene, negatively regulates the accumulation of lycopene by converting lycopene to β-Carotene in watermelon flesh. The natural missense mutation of this gene (*ClLCYB^red^*, *ClLCYB^white^* and *ClLCYB^yellow^*) causes the flesh of watermelon to show red, white and yellow colors, respectively. Further studies have indicated that these mutants would affect the ubiquitination degradation level of the ClLCYB protein by reducing the abundance of the ClLCYB protein, and further leading to the difference in the accumulation of lycopene in the flesh of watermelon fruit during ripening. In addition, Cai et al. [71] found that SlREM1 regulates the biosynthesis of lycopene via the degradation of the ubiquitin–26S proteasome pathway.

Anthocyanins are a class of important secondary metabolites in plants and important pigment components in the coloration process of many fleshy fruits such as apples, sweet cherries, grapes, etc. [115]. Anthocyanin biosynthesis is regulated by a series of transcription factors, such as BBX, HY5, MYB, bHLH, WD-repeat, etc. Among them, MYB-bHLH-WD-repeat complex plays a key role in the regulation of anthocyanin biosynthesis [116]. The ubiquitin–26S proteasome pathway plays an important role in the regulation of anthocyanin biosynthesis. In apple fruit, MdMIEL1 (MYB30-INTERACTING E3 LIGASE 1) can play a role as RING-type E3 ubiquitin ligase, and negatively regulate anthocyanin accumulation by interacting with anthocyanin-positive regulator MdMYB1 and MdMYB308L, respectively, and mediating their degradation via the ubiquitin–26S proteasome pathway [117,118]. Light is a significant environmental factor impacting anthocyanin accumulation in fleshy fruit. Light helps to lower the level of ubiquitination of anthocyanin-positive regulatory elements including MYB and bHLH, which helps to promote anthocyanin accumulation in fleshy fruit [119]. On the other hand, in dark conditions, these transcription factors’ ubiquitination levels are elevated, and their abundance will decrease [119]. RING-type E3 ubiquitin ligase COP1, an important component of the light signal pathway, exists in the cytoplasm under light conditions, but it moves to the nucleus under dark conditions, resulting in the degradation of transcription factors related to anthocyanin biosynthesis through the ubiquitin–26S proteasome pathway [120,121]. In sweet cherry, apple, and pear fruits, COP1 can modify the anthocyanin-positive regulatorsPacMYBA, MdMYB1 and PpbHLH64 by ubiquitin under dark conditions, and it can induce their degradation through the ubiquitin–26S proteasome pathway, hence preventing the accumulation of anthocyanins in fruits [119,122,123]. In addition, the E3 ubiquitin ligase MdCIP8 protein can interact with MdCOP1 through its RING-H2 domain, and inhibit the accumulation of apple anthocyanins in a way that depends on MdCOP1 in apple fruit [124]. The nitrate responsive protein MdBT2 can target the anthocyanin-positive regulator MdMYB1, MdTCP46, MdBBX22 and degrade them through the ubiquitin–26S proteasome pathway, resulting in negative regulation of the anthocyanin biosynthesis in apple fruit [125,126,127]. A 14-3-3 protein MdGRF11, in the upstream of MdBT2, interacts with MdBT2 under the condition of nitrate deficiency, and then negatively regulates the stability of MdBT2 via the ubiquitin–26S proteasome pathway, thereby increasing the abundance of MdMYB1 and activating the biosynthesis of anthocyanins [128].

In addition to E3 ubiquitin ligase, some E2 ubiquitin binding enzymes have also been reported to be involved in the metabolism of pigment in fleshy fruits. Wang et al. [129] analyzed the whole genome of tomato fruit and found that there were 52 E2 genes in tomato, of which 6 E2 genes (*SlUBC6/8/24/32/41/42*) and *PSMD2* (encoding the 26S proteasome regulatory subunit) were directly regulated by RIN, a tomato fruit ripening regulator. Silence of *SlUBC32* or *SlUBC41* will lead to the color change in tomato fruit at the mature stage. Li et al. [130] found that E2 ubiquitin binding enzyme FaUBC76 can activate the anthocyanin biosynthesis pathway to promote anthocyanin accumulation during strawberry fruit ripening. Other studies have demonstrated that DUBs can also participate in the process of pigment metabolism in fruits. For example, SlAMSH3 can regulate the metabolism of chlorophyll, lycopene and β-carotene in tomato fruit [131].

### 4.3. The Ubiquitin–26S Proteasome Pathway Is Involved in Regulating Softening of Fleshy Fruits

Softening is the sign of maturity of most fleshy fruits and the result of degradation of fruit cell wall [132]. The fruit cell wall is mainly composed of pectin, cellulose and hemicellulose, which are usually degraded by the cell wall degrading enzymes such as pectin lyase, polygalacturonase and pectin methylesterase [133]. Some studies have demonstrated that the degradation of the cell wall is also regulated by the ubiquitin–26S proteasome pathway, but the specific regulatory mechanism is still unclear [102,106,130].

Tan et al. [102] have identified 765 *E3* genes in the genomes of two peach varieties with different softening types, MF and SH, of which 515 genes (67.32%) were expressed in the flesh and 231 genes (30.20%) were differentially expressed in the flesh of the two varieties, indicating that these *E3* genes may participate in the softening process of peach fruit through the ubiquitin–26S proteasome pathway. In addition, E2 ubiquitin-binding enzyme FaUBC76 in strawberry can activate the metabolic pathway of the cell wall, while FaUBC78 plays an inhibitory role, indicating that different E2 components that mediated the ubiquitin–26S proteasome pathway may have different regulatory roles in fleshy fruit softening [130].

### 4.4. The Ubiquitin–26S Proteasome Pathway Is Involved in Regulating the Formation of Nutrient Quality of Fleshy Fruits

Fruit ripening is an important stage in the formation of fruit nutritional quality, involving the metabolic changes in various nutrients, such as soluble sugar, starch, ascorbic acid, flavonoids, organic acids, lipids, amino acids, etc., [134]. The ubiquitin–26S proteasome pathway is involved in the formation of nutritional quality during the ripening of fleshy fruits.

Wang et al. [135] found that *MaUCE1* encodes E2 ubiquitin binding enzyme in bananas, and its expression is significantly increased in the late fruit ripening period. At the same time, the activity of starch phosphorylase in fruits is also gradually increased, and the starch content is gradually decreased, which indicates that MaUCE1 may participate in starch metabolism during banana fruit ripening by mediating the ubiquitin–26S proteasome pathway.

Malic acid mainly stored in vacuoles is one of the main sources of fruit acidity [136]. MdBT2 in apple can interact with MdCIbHLH1 and MdMYB73 to decrease their protein level via the ubiquitin–26S pathway, which inhibits the accumulation of malic acid and vacuolar acidification [137,138].

NF-E2-related factor 2 (Nrf2) is a major regulator of cell oxidative stress response, which can enter the nucleus and combine with antioxidant response element (ARE) to activate the expression of the antioxidant enzyme gene [139]. Under normal physiological conditions, Nrf2 can be cyclically ubiquitinated in the cytoplasm by the E3 ubiquitin ligase Kelch-like ECH associated protein-1 (Keap-1), and degraded by the ubiquitin–26S proteasome pathway to maintain a low level. Under oxidative stress, the conformation of Keap-1 changes, thus destroying the ubiquitination level of Nrf2. The recognition ability of the 26S proteasome to Nrf2 is reduced, so that Nrf2 accumulates and then enters the nucleus to combine with ARE [139,140]. Wang et al. [140] showed that neohesperidin and hesperidin, the flavonoid components in citrus fruits, can promote the expression of Nrf2 and inhibit the expression of Keap-1. Among them, hesperidin can inhibit the ubiquitination and degradation of Nrf2 by inhibiting the activity of CUL3, thereby increasing the expression level of Nrf2, causing citrus fruits to possess natural antioxidant capacity. Lu et al. [141] found that E3 ubiquitin ligases RHA2 were highly related to the accumulation of type II and IV flavonoids via correlation analysis of transcriptome and metabolome of grape fruits. Additionally, the result suggested that the ubiquitin–26S proteasome pathway could participate in the biosynthesis of flavonoids.

## 5. Conclusions and Perspectives

Although the 26S proteasome’s structure and the ubiquitin–26S proteasome pathway are currently very well understood, their functionality in fleshy fruit ripening is still limited. Existing studies have found that the ubiquitin–26S proteasome pathway can participate in regulating many aspects of the fleshy fruit ripening process, including the biosynthesis and signal transduction of ripening-related hormones, pigment metabolism, texture change and the formation of nutritional quality. However, the specific molecular basis remains to be further analyzed. In future research work, exploring the important components of the ubiquitin–26S proteasome pathway and revealing their functional mechanism in the process of fleshy fruit ripening will help improve the regulatory network of fleshy fruit ripening, and it will provide a theoretical basis for cultivating fruit varieties with excellent ripening characteristics.

## Data Availability

The original contributions presented in the study are included in the article, further inquiries can be directed to the corresponding author.
